# Nanotherapeutic Strategies for Overcoming the Blood–Brain Barrier: Applications in Disease Modeling and Drug Delivery

**DOI:** 10.1021/acsomega.5c02206

**Published:** 2025-07-24

**Authors:** Esen Kirit, Cemile Gokce, Buse Altun, Açelya Yilmazer

**Affiliations:** † Department of Biomedical Engineering, 37504 Ankara University, Golbasi, Ankara 06830, Turkey; ‡ Stem Cell Institute, Ankara University, Balgat, Ankara 06520, Turkey

## Abstract

The blood–brain barrier (BBB) is the main obstacle preventing access to the central nervous system (CNS). It is therefore a major challenge in CNS studies, e.g., investigations of novel therapeutic agents for brain tumors, such as glioblastoma multiforme (GBM). Ensuring the structural and functional integrity of the BBB is essential for such studies. Therefore, the BBB and blood–brain-tumor barrier (BBTB) behaviors must be further investigated to enhance the treatment effectiveness in neurodegenerative diseases (NDDs). Researchers are striving to use nanoparticles (NPs) and/or develop nano delivery systems (NDSs) to efficiently overcome the barriers to transporting neurotherapeutics to the brain, focusing on targeting disease or tumor sites. In this regard, BBB disease modeling enables examination of the transport of these molecules and/or systems from the bloodstream to the brain. Facilitating their transport is likely to enhance their investigation in CNS studies and potentially lead to their use in treating various NDDs. This review describes the BBB, NPs, and/or NDSs used in BBB studies and evaluates the ability of existing BBB disease models to precisely forecast the in vivo efficacy of NPs or NDSs.

## Graphical abstract

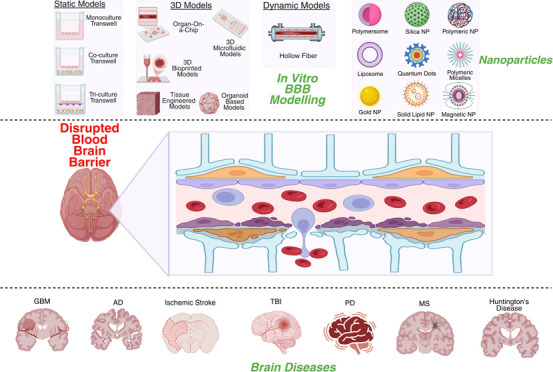

## 1 Introduction

### 1.1 The Blood–Brain Barrier

The blood–brain barrier (BBB) is a dynamic, semipermeable, multifunctional interface essential for preserving central nervous system (CNS) homeostasis by controlling molecular transportation between blood vessels and brain tissue.^1^ It is considered as a barrier in the CNS system that regulates the transport of molecules, and/or cells into and out of the brain.^2,3^ The anatomical structure of the BBB is formed depending on its functionality, and the BBB forms an important protective structure and a barrier to therapeutic drug delivery.

The BBB maintains the homeostasis of the brain by acting as a physical barrier comprising members of the neurovascular unit (NVU), which includes brain microvascular endothelial cells (BMECs), pericytes, astrocytes, basement membrane components such as tight junction (TJ) proteins (TJPs); and immune cells such as microglia.^4^ The BBB is a transport barrier based on several pathways/mechanisms, and a metabolic barrier based on different cell types, proteins, and enzymes.^5–8^ BMECs form the inner lining of the cerebral vasculature and are effectively sealed by TJPs, including claudins (e.g., CLDN5), occludin (OCLN), and junctional adhesion molecules (JAMs).^5,9^ These junctional complexes impede paracellular diffusion and generate increased transendothelial electrical resistance (TEER), thus limiting molecular transport across the barrier.^10^

Astrocytes encapsulate the vascular surface with their end-feet, controlling water and ion homeostasis through aquaporin-4 (AQP4) and other channels, while emitting regulatory chemicals like TGF-β and GDNF, which control TJPs and increase BBB integrity. Pericytes, placed in the basement membrane, modulate endothelial cell proliferation, regulate capillary blood flow, and facilitate angiogenesis. They also contribute to the composition of the extracellular matrix (ECM) by synthesizing collagen and fibronectin.^11–13^ The basement membrane includes a bilayer ECM abundant in laminins, collagen IV, nidogen, and heparan sulfate proteoglycans, providing structural support and function as signaling platforms for cellular communication and adhesion.^4,14^ The interaction between BMECs and ECM components is crucial for the polarization and barrier function of endothelial cells. Microglia, innate immune cells of the CNS, actively modulate BBB permeability. Under standard environments, they enhance barrier integrity; nevertheless, upon activation, they can produce inflammatory cytokines and reactive oxygen species, which damage TJPs and increase BBB permeability.^15,16^

The BBB exhibits heterogeneity throughout the CNS. Regional heterogeneity has been discovered, with capillaries and venules exhibiting unique profiles of transporter genes and immune modulatory proteins. The discovered heterogeneity suggests BBB function differs based on anatomical location and disease state, transporting significant implications for targeted CNS therapy (Figure 1).^17^

**Figure 1:**
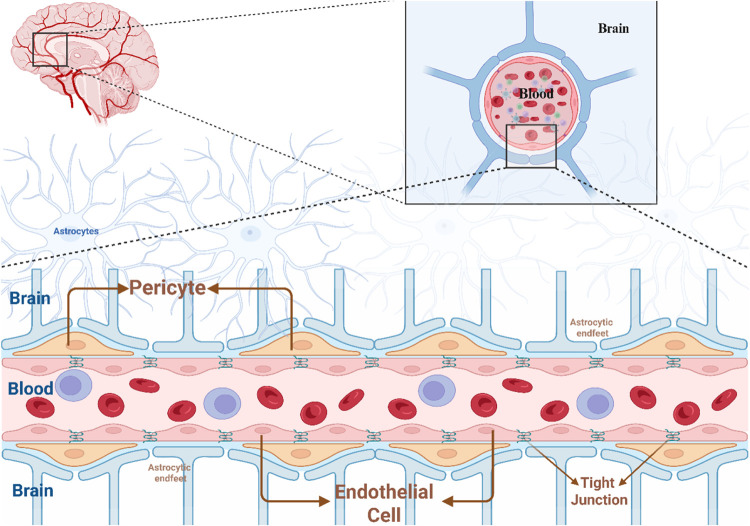
Anatomical and cellular composition of the blood–brain Barrier (Created in BioRender. Kirit,E. (2025) https://BioRender.com/qq95wsq).

The BBB offers three fundamental levels of protection. The physical barrier is constituted by TJPs, including CLDN5, OCLN, and JAMs, which tightly connect BMECs and restrict paracellular diffusion. The transport barrier depends on selective influx and efflux transporters, including P-glycoprotein (P-gp), breast cancer resistance protein (BCRP), and numerous multidrug resistance-associated proteins (MRPs), to regulate the transport of molecules over the barrier. The metabolic barrier encompasses detoxifying enzymes, including monoamine oxidases and cytochrome P450s, which neutralize potentially hazardous chemicals before their impact on brain function.^12,18,19^ However, in certain CNS illnesses, the anatomical and functional integrity of the BBB is disrupted. This disruption usually presents as a breakdown of TJPs, the loss of pericyte and astrocyte support, endothelial activation, and enhanced paracellular permeability, permitting the passage of immune cells and damaging substances into the brain.^20–23^ Such modifications are observed across an extensive range of neurodegenerative diseases (NDDs). Table 1 provides a comprehensive list of in vitro BBB models of disease, and mechanism of BBB disruptions.

**Table 1:** BBB Disruption across Neurological Diseases^a^

disease	mechanism of BBB disruption	primary consequences	refs
MS	T-cell infiltration, cytokine release, TJP loss	demyelination, lesion formation	^20,40^
NMOSD	AQP4-IgG + GRP78-Ab entry via compromised BBB	astrocyte cytotoxicity, inflammation	^41,42^
MOGAD	GRP78-Ab facilitated MOG-IgG crossing	perivenular demyelination	^26^
AE	autoantibodies pass the BBB, target synaptic receptors	neuropsychiatric symptoms	^27^
AD	pericyte loss, LRP1 downregulation	impaired Aβ clearance, chronic inflammation	^43,44^
PD	transporter dysregulation, endothelial stress	toxin accumulation, neuron loss	^45,46^
ALS	astrocyte/endothelial damage	accelerated neurodegeneration	^47,48^
stroke	ROS, MMP activation, EC death	edema, infarct expansion	^49,50^
TBI	mechanical shear, tight junction breakdown	edema, immune cell influx	^51,52^
epilepsy	seizure-induced TJ breakdown, albumin leakage	BBB leakage, enhanced excitability	^53,54^
NPSLE	immune complex + complement activation	cognitive, behavioral dysfunction	^55–57^
GBM	neovascular leakiness + partial BBB integrity	impaired drug access, therapeutic resistance	^58,59^

a AD: Alzheimer’s disease; AE: Autoimmune encephalitis; ALS: Amyotrophic lateral sclerosis; AQP4-IgG: Aquaporin-4 immunoglobulin G; GBM: Glioblastoma multiforme; GRP78: Glucose-regulated protein 78; MS: Multiple sclerosis; NMOSD: Neuromyelitis optica spectrum disorder; PD: Parkinson’s disease; TBI: Traumatic brain injury; TJPs: Tight junction proteins.

In inflammatory degenerative diseases such as multiple sclerosis (MS) and neuromyelitis optica spectrum disorder (NMOSD), BBB failure enables immune cell infiltration and enhances the activity of autoantibodies such as AQP4-IgG and GRP78, resulting in neuroinflammation and demyelination.^20,24,25^ In MOGAD, the same processes facilitate the entrance of MOG-IgG into the CNS.^26^

In autoimmune encephalitis (AE), BBB permeability increases and allows the passage of neuronal autoantibodies that damage synaptic function.^20,27^ NDDs such as Alzheimer’s disease (AD) and Parkinson’s disease (PD) are distinguished by gradual BBB degradation, transporter dysregulation, and decreased clearance of hazardous substances.^1,28–30^ Amyotrophic lateral sclerosis (ALS) exhibits BBB damage resulting from astrocytic and endothelial dysfunction.^31^

Acute disorders, such as stroke, traumatic brain injury (TBI), and epilepsy, produce acute barrier disruption due to oxidative stress, mechanical injury, or seizure activity, leading to edema and inflammatory damage.^32–34^ In neuropsychiatric lupus (NPSLE), the deposition of antibodies and activation of complement damage the BBB and lead to cognitive and psychiatric symptoms.^35,36^

In addition to its biological benefits, such as safeguarding the brain, the BBB presents an important obstacle for medical treatments by limiting the entry of most pharmaceuticals into the CNS. This restriction is most apparent in glioblastoma multiforme (GBM), where abnormal neovascularization leads to various and partly dysfunctional BBB areas.^37^ Although some permeable zones may permit restricted drug penetration, other areas remain unchanged, leading to irregular drug distribution, decreased effectiveness, and eventually, therapeutic resistance. Consequently, the BBB should be regarded as an essential component in the development of targeted approaches for the treatment of NDDs and brain malignancies, including GBM.

With the development of nanomedicine, the use of NDSs as a treatment modality for NDDs such as AD and BC has gained importance. Although conventional treatment methods, such as chemotherapy, have substantial adverse effects, and the passage of drugs across the BBB is restricted, these methods continue to be considered the most effective cancer treatments, particularly for BC. Therefore, new strategies must be developed with unique physicochemical properties, low cytotoxicity, and high functionality for drug transport; diminished adverse effects and treatment resistance; lower dosing; and traceable therapeutic agents that can cross the BBB.^38,39^ This article first aims to provide a brief review of the BBB. Subsequently, various BBB models of NDDs in the literature that use NDSs for multiple purposes, such as understanding NP efficiency and BC treatment, are described, and their challenges are discussed.

### 1.2 In Vitro Modeling of BBB

The in vitro modeling of the BBB has advanced considerably, evolving from simple monoculture experiments to complex, multicellular, and dynamic systems. These advances aim to replicate the complex structure of the NVU and its interconnections in both healthy and diseased conditions. As shown in Figure 2, recent BBB models are classified as static (e.g., Transwell), dynamic (e.g., microfluidic), organoid-based, and tissue-engineered platforms, each presenting distinctive benefits and drawbacks for nanotherapeutic assessment and CNS disease modeling.^60,61^ For further information and recent examples of these models, readers can refer to other review articles.^62–68^

**Figure 2:**
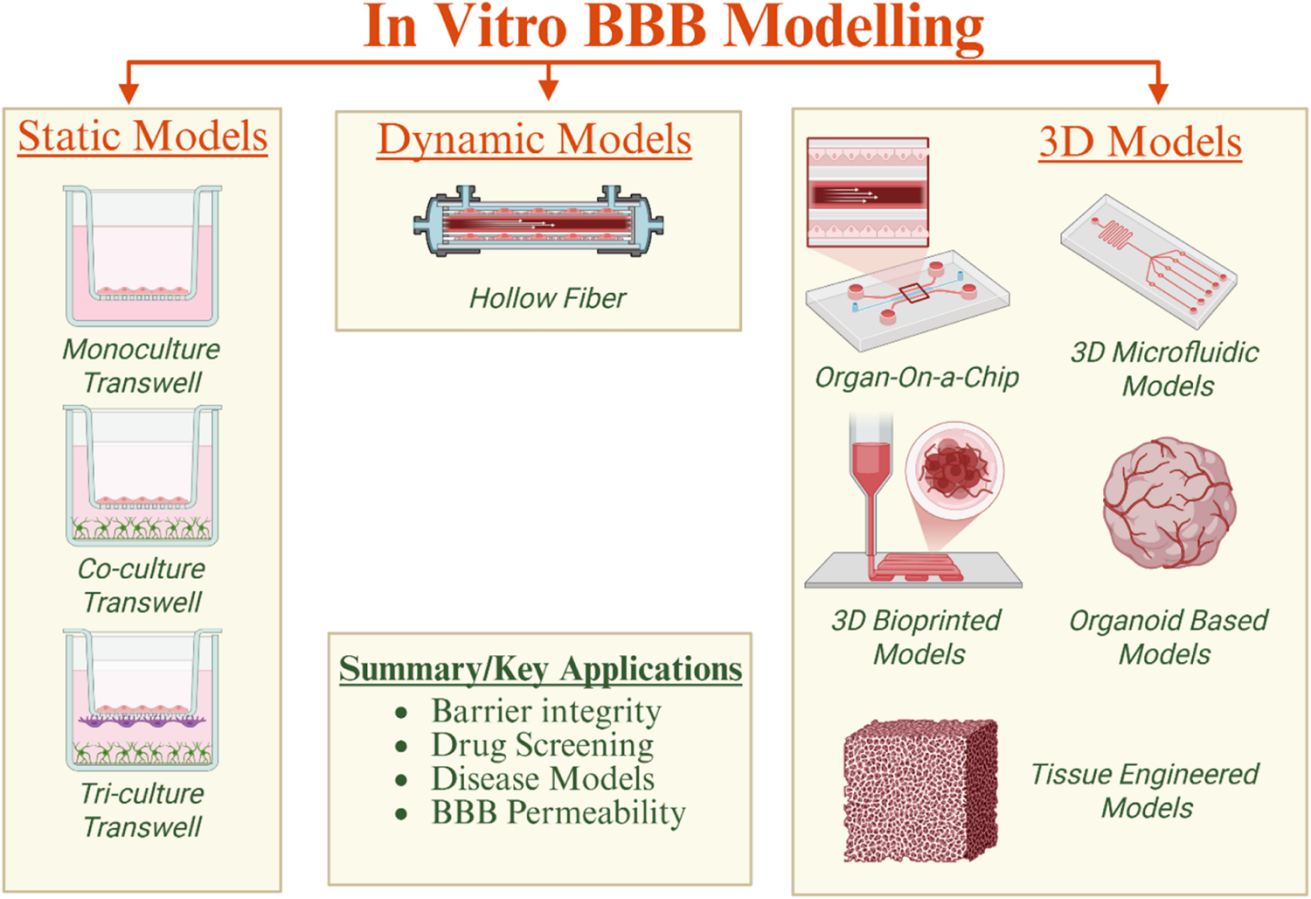
In vitro BBB modeling (Created in BioRender. Kirit, E. (2025) https://BioRender.com/im7gbyr).

### 1.3 Studying Diseases with BBB Models

The integrity of the BBB is modified and disrupted in various NDDs such as AD, Huntington’s disease, and PD.^69^ The types and stages of CNS diseases affect both the function and integrity of the BBB, and influence drug delivery across the BBB and the therapeutic efficacy against the disease.^13,16^ Because of changes in both BBB structure and function, developing BBB models with diseases is critical for understanding barrier disruption, dysfunction, and pathological proteins in drug delivery and disease therapy studies.^1,70,71^ Conventional drug delivery systems are generally ineffective because they are unable to cross the BBB. However, advances in research have enabled the development of nanotheranostics. The BBB can be penetrated by some nanoparticles (NPs).^72,73^ In this context, researchers continue to innovate in the field of nanomedicine by developing various types of nanosystems. Because of the expense of testing nanoformulations in vivo, most initial studies on nanoparticle formulation are performed in vitro, in relevant cell models such as endothelial cells, neuronal cells, or glial cells.^74^ Table 2 provides a comprehensive list of the cell types used to establish in vitro BBB models of disease, as well as examples of the nanoparticle formulations tested in these models.

**Table 2:** BBB Disease Models in Nanomedicine^a^

targeted disease	study type	nanosystem	BBB model type	targeted cells	refs
Parkinson’s disease	in vivo and ex vivo	melatonin/polydopamine nanostructures (mPDAN)	3D organoid	SH-SY5Y and IMR-32 cells (human neuroblastoma cell lines)	^81^
	in vivo	dopamine-loaded blood exosomes	transwell	bEnd.3 cells (mouse brain microvascular endothelial cell line)	^82^
	in vivo	Iron oxide nanoparticles (IONPs)	transwell	bEnd.3	^83^
	in vitro	selenium nanoparticle (SeNP)-loaded l-DOPA/dopamine	transwell	hBEC-5i cells (human brain microvascular endothelial cell line)	^77^
	in vivo	mesoporous silica-encapsulated gold nanorods (MSN-AUNRs)	transwell	bEnd.3 cells	^84^
multiple sclerosis	in vivo	curcumin-loaded HPPS nanoparticles	transwell	monocytes	^85^
Huntington’s disease	in vitro	MnFe_2_O_4_ nanoparticles	transwell	bEnd.3 cells	^86^
	in vitro	cyclodextrin nanoparticles (CDs) loaded with small interfering RNAs	transwell	hCMEC/D3 cells (human brain endothelial cell line)	^78^
Alzheimer’s disease	in vivo	transferrin-modified Ost liposomes (Tf-Ost-Lip)	transwell	APP-SH-SY5Y cells	^87^
	in vitro	peptide functionalized hollow gold nanospheres and gold nanorods	microporous polycarbonate membrane filters	PBCE cells (porcine brain capillary endothelial cells)	^87^
	in vivo	nanoparticles (FTY@Man NP) constructed from a PLGA–PEG skeleton loaded with fingolimod (FTY) and externally modified with mannose	transwell	bEnd.3 and BV-2 cells (mouse microglial cell line)	^88^
	in vivo	protoporphyrin IX (PX)-modified oxidized mesoporous carbon nanospheres (OMCN)	transwell	SH-SY5Y cells	^89^
	in vivo	MEM–PEG–PLGA nanoparticles (NPs)	transwell	bEnd.3 cells and astrocytes	^90^
	in vitro	gold nanorod (GNR)-PEG-Ang2/D1	BBB-on-a-chip	human hippocampal astrocytes, human brain-vascular pericytes, and hCMEC/D3 (human brain endothelial cell line)	^91^
	in vitro	sialic acid (SA)-modified selenium (Se) nanoparticles	transwell	bEnd.3 cells	^92^
stroke	in vitro	edaravone loaded ceria nanoparticles (E-A/P-CeO_2_)	transwell	BCECs (brain capillary endothelial cells)	^93^
	in vivo	Neuroprotectant (ZL006) loaded liposomes (T7 and SHp-P-LPs/ZL006)	transwell	BCECs	^94^
	in vitro	Fe_3_O_4_ nanoparticles (MNP) loaded with dexamethasone (dm@LMNP)	transwell	BCECs	^95^
many neurological diseases	in vivo	Tf-containing gold nanoparticles (AUNPs)	transwell	bEnd.3 cells	^96^

a Ang2: Angiopep-2.

Polylactide (PLA) NPs have been used as drug carriers for the delivery of flurbiprofen, an Aβ42-lowering drug, across the BBB in the context of AD. The Aβ42 peptide has been implicated in AD pathogenesis and drug development for AD, but has faced challenges of poor BBB penetration.^75^ Flurbiprofen has been encapsulated in PLA NPs, which might serve as effective carriers for delivering flurbiprofen across the BBB and modulating Aβ42 levels; this treatment holds promise in the development of novel AD therapies.^76^ Another study has focused on selenium NPs (SeNPs), because of their small size, biocompatibility, low toxicity, easy preparation, and photoreactive, anticancer, and biocidal properties.^77^ The study has described the PAMPA-BBB acellular assay as an appropriate model for predicting cross-BBB permeability. That study used nontoxic doses of SeNP for the active transport mechanism in combination with l-DOPA or dopamine for BBB permeability studies. In vitro evaluation with cell-free and cellular transwell models has demonstrated that SeNP-loaded l-DOPA/dopamine is effectively internalized by human brain ECs, thus highlighting the potential of SeNPs to serve as drug delivery vehicles in PD therapy (Figure 5).^77^ An innovative, noninvasive gene therapy delivery system has been developed to treat Huntington’s disease, a neurodegenerative disease caused by a mutation of the huntingtin (HTT) gene. Gene therapies aimed at decreasing levels of the mutant HTT protein have shown promise. Cyclodextrin (CD)-based nanoparticles carrying small interfering RNAs (siRNAs) have been functionalized with rabies virus glycoprotein (RVG) to facilitate crossing of the BBB. Human cerebral microvascular endothelial cells (hCMEC/D3) and rat striatal neuronal cells (ST14A) expressing a mutant HTT gene have been used in in vitro models. These CD nanoparticles successfully cross the BBB, release siRNAs into neuronal cells, and efficiently decrease HTT gene expression. Furthermore, the integrity of the BBB model is maintained, and the nanoparticles do not damage endothelial cells. This CD-based delivery system is a notable noninvasive and effective platform that might potentially increase the applicability of siRNA therapies to Huntington’s disease.^78^ Ischemic stroke is an acute cerebrovascular disease with substantial mortality and disability rates. However, the clinical application of neuroprotective drugs remains limited by challenges such as poor BBB penetration and rapid drug inactivation in circulation. Nanomaterial-focused therapeutic studies have been reviewed previously.^79^ Nanomaterials have therapeutic potential in treating ischemic stroke, facilitating drug delivery, increasing drug bioavailability, achieving long drug half-lives, and enhancing dissolution. A novel in vitro BBB penetration model has been used to evaluate the efficacy of BBB-targeted lipid nanoparticles (T-LNPs) in the delivery of Ferrostatin-1 (Fer1), a ferroptosis inhibitor. With a mouse brain microvascular endothelial cell (bEnd3) monolayer, T-LNPs, compared with nontargeted nanoparticles, show significantly enhanced penetration under oxygen-glucose deprivation conditions mimicking ischemia. Encapsulation of Fer1 in T-LNPs not only facilitates BBB penetration but also bioactivity retention, thus achieving more favorable inhibition of ferroptosis, decreased oxidative stress, and greater neuroprotection in ischemic stroke models. These findings underline the efficacy of the in vitro BBB model in developing drug delivery strategies and highlight T-LNPs as a promising approach to overcome BBB-related challenges in stroke therapy.^80^

### 1.4 Nanoparticles Crossing the BBB

The number of cases of CNS diseases, particularly AD, PD, brain tumors, and strokes, is growing; these diseases currently rank second among fatal diseases in terms of numbers of deaths.^97,98^ Nevertheless, the efficacy of CNS drug development is extremely low.^99^ Initial research has shown substantial promise for nanomedicines in CNS disease treatment. Nanotechnology has been extensively used in the field of drug delivery and has shown potential to significantly enhance the effectiveness and efficiency of drugs.^100–102^ A wide range of nanocarriers, such as polymeric NPs, inorganic NPs, liposomes, nanofibers, and micelles, have been designed to deliver therapeutic and diagnostic substances.^103,104^ In Table 3 an extensive comparison of nanoparticle is shown. NPs can transport medications across the BBB and have effects on the CNS. Through changes and including chemical constituents, NPs can effectively cross the BBB and access specific locations within the brain. This ability is crucial for delivering drugs to the brain. These particles avoid phagocytosis by the reticuloendothelial system, thereby enhancing drug delivery to the brain by allowing pharmaceuticals to cross the BBB and significantly increasing the drug amounts present in the brain. This aspect is crucial in both theoretical and clinical studies on drug delivery systems.^105^

**Table 3:**
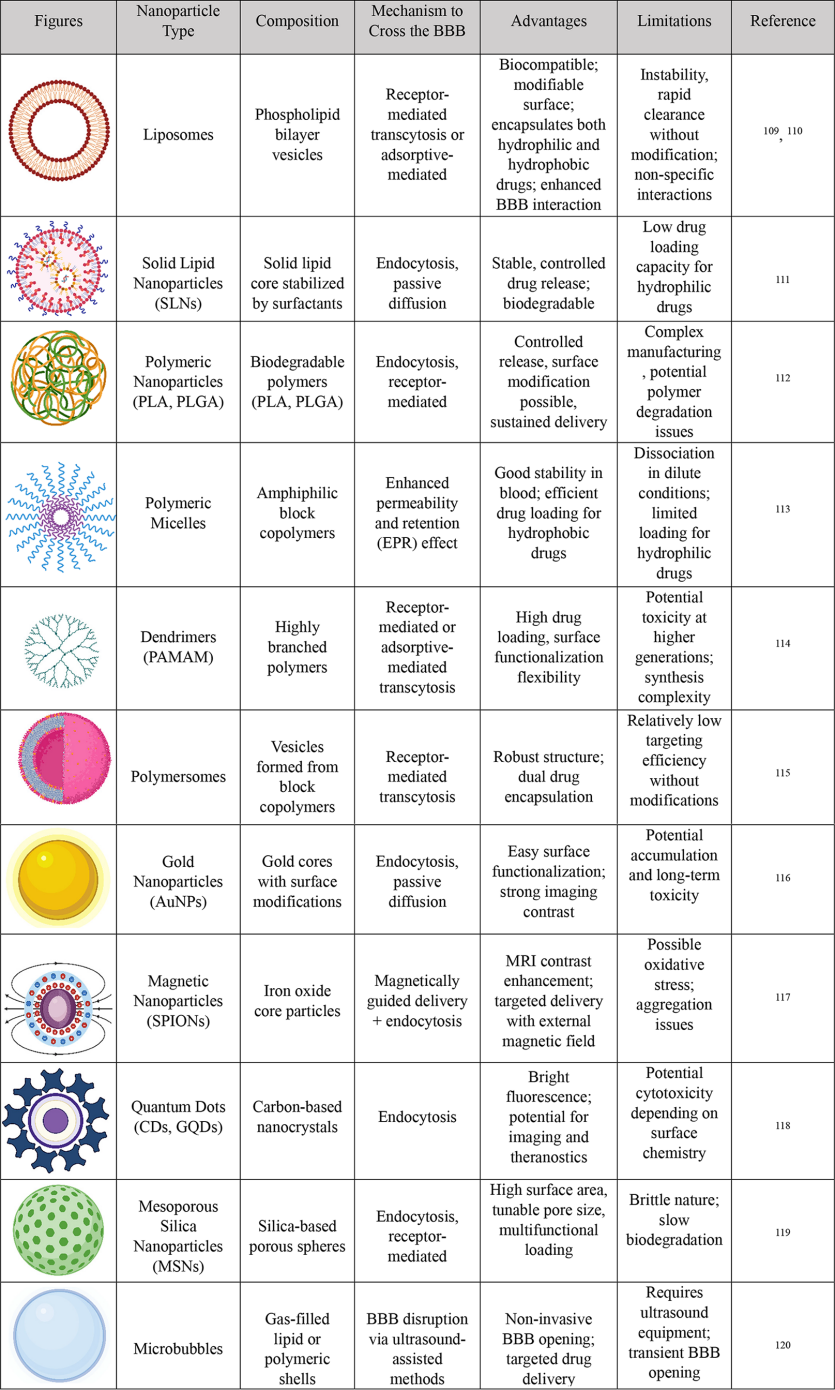
Comparison of Nanoparticles

Size, a critical factor determining the behavior of NPs, affects NPs’ ability to deliver drugs to specific tissues, be taken up by cells, and interact with target proteins. Even chemical reactions involving NPs are influenced by NP size. The precise location and nature of the targeted tissue also influence the ideal NP size. For instance, most cancers have a vascular pore cutoff size ranging from 380 to 780 nm.^106^ NPs are solid colloidal particles consisting of a polymer or lipid. The particle size varies from 10 to 1000 nm, and typically ranges from 50 to 300 nm.^107^ NPs are ideal for drug targeting to the BBB.

NPs encounter obstacles when crossing the BBB and entering the brain. To address these issues, many techniques have been developed to improve NP transport across the BBB by exploiting the physiological mechanisms involved. A frequently used technique involves inducing a transient increase in BBB permeability via the paracellular pathway, often by breaking the tight junctions between nearby ECs; to do so, ultrasound with microbubbles or osmotic pressure can be used to locally increase BBB permeability and enhance NP entry.^108^ Other methods of transporting NPs include receptor-mediated transcytosis, adsorptive transcytosis, carrier-mediated inflow, efflux mechanisms, and the paracellular aqueous diffusion pathway.

NP-based drug carriers must meet the following requirements: (1) they must be nontoxic, biodegradable, and biocompatible; (2) they must have a particle diameter less than 100 nm; (3) they must be stable in the bloodstream and not have a tendency to aggregate; (4) they must have a streamlined and economical production process; and (5) they must be resistant to uptake by the mononuclear phagocytic system, thus preventing any conditioning effects and displaying prolonged circulation times in the blood. Several NP-based formulations are now under evaluation for the treatment of CNS diseases, as summarized below. In Tables 4 and 5 shows various nanoparticle treatments in preclinical and clinical studies.

**Table 4:** Preclinical Studies on NPs for Brain Tumor Treatment^a^

study type	nanomaterial/nanoparticle/nanodelivery system	refs
in vitro and in vivo	liposomes + DOX + CB5005	^125^
	liposomes + paclitaxel (PTX) + Rg3	^176^
in vitro	liposomes + super paramagnetic iron oxide NPs (SPIONs) + DOX + P1NS + TNC	^177^
in vitro and in vivo	liposomes + rapamycin + MTI-31 + VAP	^178^
	siPLK micelle + TMZ + Angiopep-2 (Ang2)	^179^
	micelle + platinum + cyclic RGD (cRDG)	^180^
in vitro	dendrimer (PAMAM) + DOX + Ang2	^181^
	PAMAM + arsenic trioxide + cRGD	^182^
in vitro and in vivo	PAMAM + PEG+ TRAIL + transferrin (Tf)	^152^
in vitro	PAMAM + tamoxifen + DOX + PEG + Tf	^183^
in vitro and in vivo	cyclodextrin + butylidenephthalide	^184^
	magnetic double emulsion nanocapsules + lactoferrin	^185^
	spherical nucleic acid NPs (gold NPs)	^186^
in vitro	SPION + HAPtS	^187^
in vitro and in vivo	silver (Ag) NPs + verapamil + AS1411	^188^
	iron oxide NPs + cisplatin + folic acid	^189^
	poly(butylcyanoacrylate) (PBCA) NPs + cisplatin	^190^
	PLGA NPs + methotrexate/PTX + poloxamer 188	^191^
	BSA NPs + DOX + PEG + lactoferrin	^192^
	Ph-dye NPs + ApoE	^193^
	poly(levodopamine) NPs + DOX + indocyanine green	^194^
	Ag–In–S ternary quantum dots + cysteine + KLA	^195^
	carbon nitride dots	^196^
in vitro and in vivo	graphene quantum dots	^168,197^
in vitro and in vivo	carbon dots (CDs)	^169,198–200^
in vitro	CDs + DOX + Tf	^201^
in vitro and in vivo	iron-doped orange emissive CDs	^202^
	boron CDs + exosome	^118^
	sugar CDs	^203^
in vitro	CDs + Pep1 + epirubicin + TMZ	^204^

a ApoE: Apolipoprotein E; CDs: Carbon Dots; DOX: Doxorubicin; PTX: Paclitaxel; TNC: Tenascin-C; MTI-31: Mitochondrial targeting inhibitor 31; VAP: Vascular adhesion protein; cRGD: cyclic RGD peptide; PAMAM: Poly­(amidoamine) dendrimers; PEG: Poly­(ethylene glycol); Tf: Transferrin; HAPtS: Hydroxyapatite shell; PBCA: Poly­(butylcyanoacrylate); PLA: Poly­(lactic acid); PLGA: Poly­(lactic-*co*-glycolic acid).

**Table 5:** NPs Are Clinically Used in Brain Tumor Treatment^a^

national clinical trial number	tumor type	nanoparticle/nanodelivery system
NCT02340156	GBM	liposomes + temozolomide (TMZ) + SGT-53
NCT01906385		liposomes + rhenium 186
NCT02861222	refractory nonbrainstem malignant glioma	liposomes + doxorubicin (DOX)
NCT00019630	refractory solid tumors	liposomes + HCL+ DOX
NCT01848652	relapsed and refractory primary CNS lymphoma	liposomes + PEG + DOX
NCT03328884	brain metastases	liposomes + irinotecan (CPT-11)
NCT03086616	diffuse intrinsic pontine glioma	convection-enhanced delivery of liposomes + irinotecan
NCT03818386	brain metastases	gadolinium-based NP (AGuIX)
NCT04881032	GBM	AGuIX + TMZ
NCT03020017		gold NPs + NU-0129
NCT03463265		albumin-based NPs + rapamycin
NCT03250520	brainstem glioma	platinum acetylacetonate + titanfia (NPt-Ca)

a AGuIX: Gadolinium-based nanoparticle.

#### 1.4.1 Lipid-Based Nanoparticles

##### 1.4.1.1 Liposomes

Liposomes, the first-generation nanoparticulate drug delivery systems,^109^ include several vesicular bilayers (lamellae) composed of amphiphilic lipids that encapsulate an interior aqueous compartment. The liposomal lipid bilayer typically consists of biocompatible and biodegradable lipids that are found in biological membranes. Key components of liposomes include sphingomyelin, phosphatidylcholine, and glycerophospholipids. Because of its ability to decrease membrane permeability and increase liposome stability in living organisms, collagen is frequently incorporated into liposomes.^121^ One beneficial characteristic of liposomes is their amphiphilic properties, which allow for free diffusion through cell membranes and targeting of brain cancer cells.

Liposome carrier systems have received approval from the Food and Drug Administration for clinical use. This milestone was achieved with the approval of Doxil (doxorubicin hydrochloride liposome injection) produced by Sun Pharma Global FZE, based in Mumbai, India.^122–124^ The study focused on improving glioma treatment with CB5005-modified PEGylated liposomes. CB5005, a cell-penetrating peptide and NF-κB inhibitor, was attached to modified liposomes. In vitro examinations demonstrated that CB5005 significantly enhanced liposomal absorption by glioma cells and elevated DOX liposome cytotoxicity against U87 tumor cells. In vivo imaging revealed that intravenous CB5005-LS accumulated in the brain and targeted GBM areas. The dual-functional CB5005-LS/DOX system significantly increased the survival rates of mice with intracranial GBM.^125^ Liposomes have frequently been used for delivering drugs to the brain to treat illnesses such as cerebral ischemia (Lai et al.). For example, Ishii et al. have discovered that FK506 liposomes efficiently repair cerebral ischemia/reperfusion injury. If administered shortly after reperfusion, these liposomes, compared with free FK506, significantly decrease neutrophil infiltration, apoptotic cell death, and infarct volume in t-MCAO rats, thereby enhancing motor function problems. Therefore, FK506 liposomes, which allow for lower dosages without loss of efficacy, have considerable potential as a neuroprotective drug if provided promptly after a stroke,^126^ and can deliver opioid peptides and target brain tumors.^127^ Liposomes coated with transferrin can cross the BBB. Mangostin, a polyphenolic xanthone that preserves cerebral cortical neurons, can be effectively transported across the BBB with transferrin liposomes. This technique, compared with individual transport, improves drug bioavailability in the plasma. Folate, epidermal growth factor (EGF) receptors, and avb3 integrin are several potential target delivery systems for liposomes, including active vascular targeting.^128^

##### 1.4.1.2 Cationic Liposomes

Cationic liposomes, which consist of lipids with a positive charge, have been created and used primarily as carriers for transfection. Their purpose is to transport genetic material, such as DNA, into cells while preventing degradation by lysosomes. The most frequently used cationic lipid is 1,2-dioleoyl-3-trimethylammonium-propane (DOTAP), which is combined with dioleoyl-phosphatidyl-ethanolamine (DOPE). Cholesterol also enhances transfection levels and may decrease liposome instability in the presence of serum.^110^ The cationic lipids and nucleic acids interact and form complexes known as lipoplexes. Positively charged liposomes adhere to the negatively charged phosphate molecules on the DNA backbone through electrostatic interactions. When the pH is lowered to 5–6, DOPE undergoes acidification and subsequently combines with and collapses the endosome membrane. Consequently, the contents of the endosome are released into the cytosol. Therefore, drugs have the potential to be transported into endothelial cells, similarly to DNA, thereby increasing their BBB crossing and targeting of neurons.^129,130^ Zhao et al. have demonstrated that lipoplexes are considerably more efficient at transferring neuronal SH-SY5Y cells than the frequently used transfectant Lipofectamine. When cationic liposomes carrying photoreactive drugs are triggered with a laser, they have lethal effects on glioblastoma cells. Furthermore, these liposomes have been found to increase the distribution of the cancer treatment drug paclitaxel to the brain in animals.^130,131^ Studies have revealed that Lipid NPs with an inherent positive charge confer advantages in drug delivery.^132^ Future research should prioritize the development of cationic NPs for the delivery of frequently used drugs.

##### 1.4.1.3 Solid Lipid Nanoparticles

Solid lipid NPs (SLNs) are lipid-based nanocarriers with a stable hydrophobic lipid core, which enable the dissolution or dispersion of drugs.^111^ They are composed of biocompatible lipids, such as triglycerides, fatty acids, or waxes. Typically, nanoparticles are modest in size, ranging from 40 to 200 nm. This size enables them to cross the tight endothelial cells of the BBB and avoid being trapped by the reticuloendothelial system.^133^ SLNs have various advantages, including biocompatibility, more favorable drug entrapment effectiveness than other nanoparticles, and the ability to achieve sustained drug release over several weeks.^113^ Wang et al. have documented the synthesis of 3,5-dioctanoyl-5-fluoro-2-deoxyuridine (DO-FUdR) to address the limited availability of the drug 5-fluoro-2-deoxyuridine (FUdR) and its integration into SLNs. The results demonstrated that DOFUdR-SLN has almost twice the brain-targeting efficacy of free FUdR in vivo. SLNs, therefore, might enhance drugs’ ability to cross the BBB and serve as a valuable system for targeting drugs to treat CNS illnesses.^134,135^ The primary mechanism involved in malignant GBM is interference with intracellular mRNA activity through the administration of siRNAs via lipid NPs.^136^ The prominence and therapeutic promise of tumor-targeted drug delivery are increasing. Given lipid NPs’ ability to efficiently cross the BBB, the cytotoxicity of these NPs must be considered.^137^ Lipid NPs outperform other nanomaterials in terms of cargo therapeutic biodistribution and bioavailability. Their limitations include particle accumulation, unpredictable behavior and unexpected changes in polymer characteristics, and sudden dissolution of the administered drugs.^138^

#### 1.4.2 Polymer-Based Nanoparticles

##### 1.4.2.1 Polymeric Nanoparticles

Polymeric NPs consist of a core polymer matrix that can incorporate medicines, typically 60–200 nm in size.^112,139^ In recent years, several polymers have been specifically engineered for medical purposes and used in the field of controlled release of bioactive substances. A substantial number of these materials have been specifically engineered to undergo degradation within the human body. The most frequently used polymers are PLA and polyglycolides. The materials described are poly­(lactide-*co*-glycolides) (PLGA), polyanhydrides, polycyanoacrylates, and polycaprolactone. Despite advancements in synthetic and semisynthetic polymers, natural polymers such as chitosan can still be used. Multiple studies have demonstrated that substances such as ANG-PEG-NP can effectively address the problem of limited permeability in the tumor blood barrier.^140^ Choonara et al. have used NPs composed of PLGA to encapsulate antituberculosis medications (rifampicin, isoniazid, pyrazinamide, and ethambutol) for targeted drug delivery to the brain. When provided to mice, these NPs achieve sustained high drug levels in the plasma for 5–8 days and in the brain for 9 days, significantly longer than the duration achieved with administration of the free drugs.^141^ Pandey et al. have reported that administering five doses of the NP formulation to mice infected with *Mycobacterium tuberculosis* leads to bacterial removal from the meninges after the administration of only 46 doses of common drugs.^142^ Functional proteins have been effectively delivered into neurons and neuronal cell lines with poly­(butylcyanoacrylate) NPs in another study.^143^ Mangraviti et al. have synthesized NPs by modifying poly­(1,4-butanediol diacrylate-*co*-4-amino-1-butanol) with 1-(3-aminopropyl)-4-methylpiperazine. These NPs have been used to deliver herpes simplex virus type I thymidine kinase (HSVtk) and ganciclovir (GCV) in a malignant glioma model (Figure 3).^144^ Nonetheless, certain polymeric NPs have higher cytotoxicity than other delivery techniques. As a result, a thorough analysis of the safety risks is essential before any polymeric NPs are used in clinical settings for the treatment of brain cancer.

**Figure 3:**
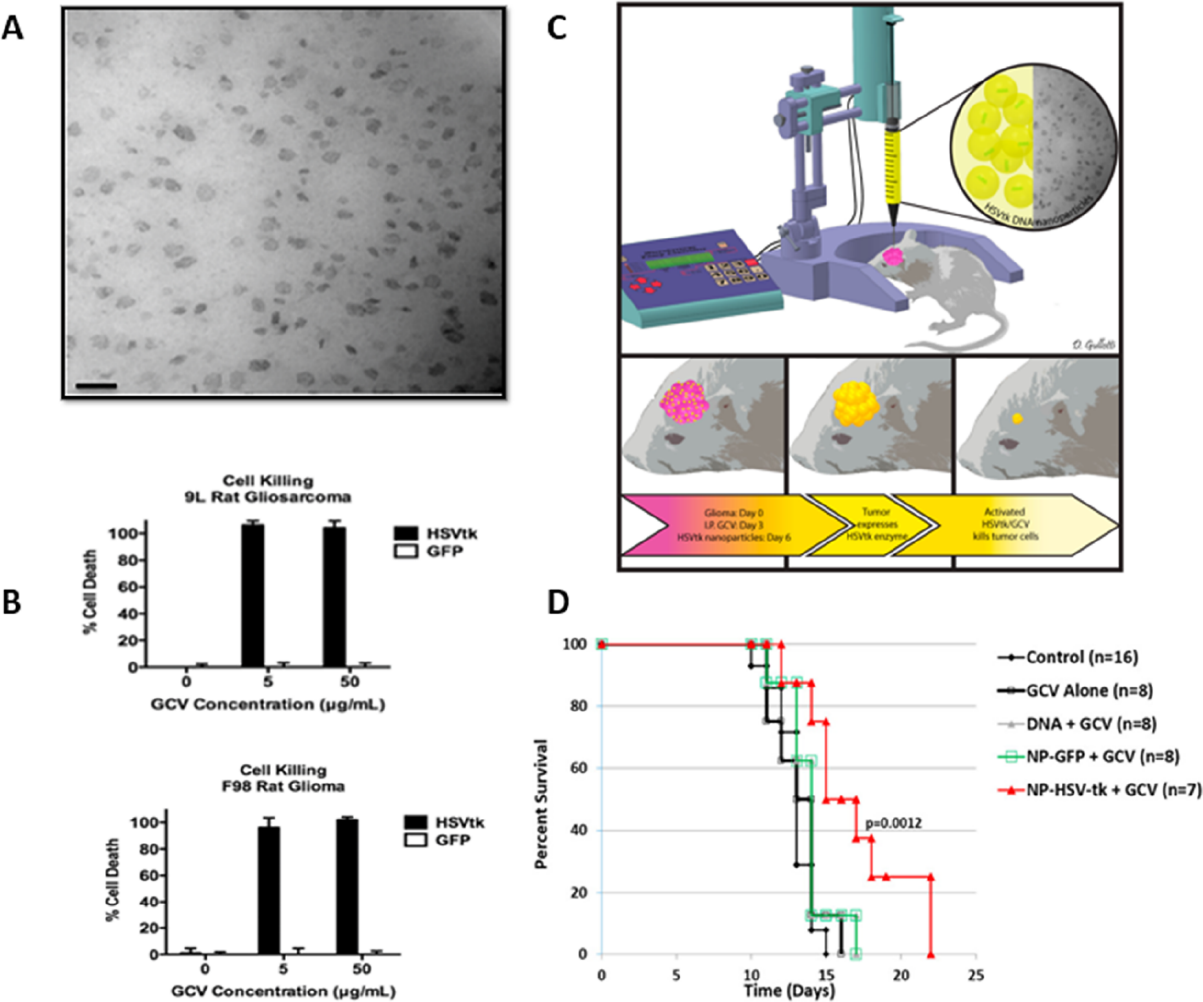
PBAE/HSVtk/GCV biodegradable polymeric NPs conferring extended survival in gliosarcoma both in vitro and in vivo. (A) TEM image of fresh PBAE NPs; scale bar: 100 nm. (B) Transfection of malignant glioma cells (9L and F98) with HSVtk and GCV elicits cancer-cell killing in vitro. (C) Summary of the in vivo study approach. (D) Plots showing substantial extension of the survival in F344 rats implanted with 9L gliosarcoma, after administration of PBAE/HSVtk/GCV. Reprinted from Mangraviti et al., Copyright American Chemical Society (2015).

##### 1.4.2.2 Polymeric Micelles

Polymeric micelles are created by amphiphilic copolymers that aggregate in aqueous solution and form spherical structures. These structures have a hydrophilic outer shell and a hydrophobic inner core and exhibit high stability 130. Polymeric micelles can respond to external stimuli. Such as pH, light, temperature, and ultrasound, thereby enabling controlled release of the pharmaceuticals contained within them.^145^ The potential of these NPs to deliver drugs to the brain has been demonstrated. For example, mice injected intravenously with chitosan-conjugated pluronic nanocarriers with a specific target peptide for the brain (rabies virus glycoprotein; RVG29) have shown accumulation of either a quantum dot (QD) fluorophore conjugated to the nanocarrier or a protein loaded into the carrier in the brain.^146^

##### 1.4.2.3 Dendrimers

A dendrimer is usually symmetrical concerning its core, and when it is sufficiently extended, it tends to adopt a spherical three-dimensional shape in water. The structure comprises a central core with a minimum of two similar chemical functional groups. From these groups, other molecules can develop, forming repeating units with at least one branching junction. The recurrence of chains and branches leads to the formation of increasingly dense concentric layers. The structure of dendrimers is densely packed at the outside edges and less densely packed at the center, thus creating gaps that are crucial for trapping drugs.^114^ These entities are classified as nanovectors, whose surface properties can be varied to enable binding to hydrophobic or hydrophilic chemicals with high molecular weight.^147^ Poly­(amidoamine), often known as PAMAM, is widely recognized as the primary chemical used for synthesizing dendrimers. The main component of PAMAM is a diamine, often ethylenediamine, which reacts with methyl acrylate before reacting again with ethylenediamine, thus producing generation-0 PAMAM. Subsequent reactions generate more advanced generations. Dendrimers are currently being investigated for a variety of medical applications, including the treatment of brain cancers with nanomedicine. However, administering medicine with dendrimers poses toxicity issues. Therefore, toxicity must be considered before dendrimers are used as a clinical delivery vector. Albertazzi et al. have discovered that the functionalization of PAMAM dendrimers has clinical effects on their ability to spread across the CNS tissue of living organisms and enter live neurons. This was observed after injection directly into the brain tissue (intraparenchymal) or the brain ventricles (intraventricular).^148^ Vidal and Guzman et al. have described a drug intended to transfer DNA into the brain by using serine-arginine-leucine (SRL) functionalized PAMAM dendrimers. The SRL peptide was associated with G4 PAMAM dendrimers via a double-functional poly­(ethylene glycol) (PEG). The modification of dendrimers with SRL led to vulnerability to clathrin/caveolin energy-dependent endocytosis in the brain capillary system, thereby improving rates of transfer and decreasing toxicity.^149^ Zhao et al. have successfully developed a dendrimer-based delivery system tailored to GBM. PEGylated CREKA-modified PAMAM dendrimers have been demonstrated to cross GBM tissue and show greater retention than untreated NPs. Therefore, modified dendrimers are highly appropriate for delivering chemotherapy drugs.^150^ Somani et al. have developed a 3-diaminobutyric polypropylenimine dendrimer coated with lactoferrin to transport a therapeutic gene into the brain (Somani et al.). Kannan et al. have demonstrated that polyamidoamine dendrimers, when supplied systemically, aggregate solely in activated microglia and astrocytes in the brains of newborn rabbits with cerebral palsy. This research might suggest the potential of clinical applications in the treatment of neuroinflammatory diseases in humans.^151^ In addition, Gao et al. have reported a gene-drug delivery system based on transferrin Tf-modified PAMAM for glioma treatment (Figure 4). The approach includes a plasmid that encodes the tumor necrosis factor-induced apoptosis-inducing ligand (trail) and produces NPs through condensation with Tf-modified PAMAM. PAMAM–PEG-Tf/DNA NPs outperform PAMAM–PEG/DNA NPs in terms of cellular uptake, in vitro gene expression, and cytotoxicity in C6 glioma cells. Ex vivo fluorescence imaging has indicated the potential of Tf-modified NPs to target certain tumors.^152^

**Figure 4:**
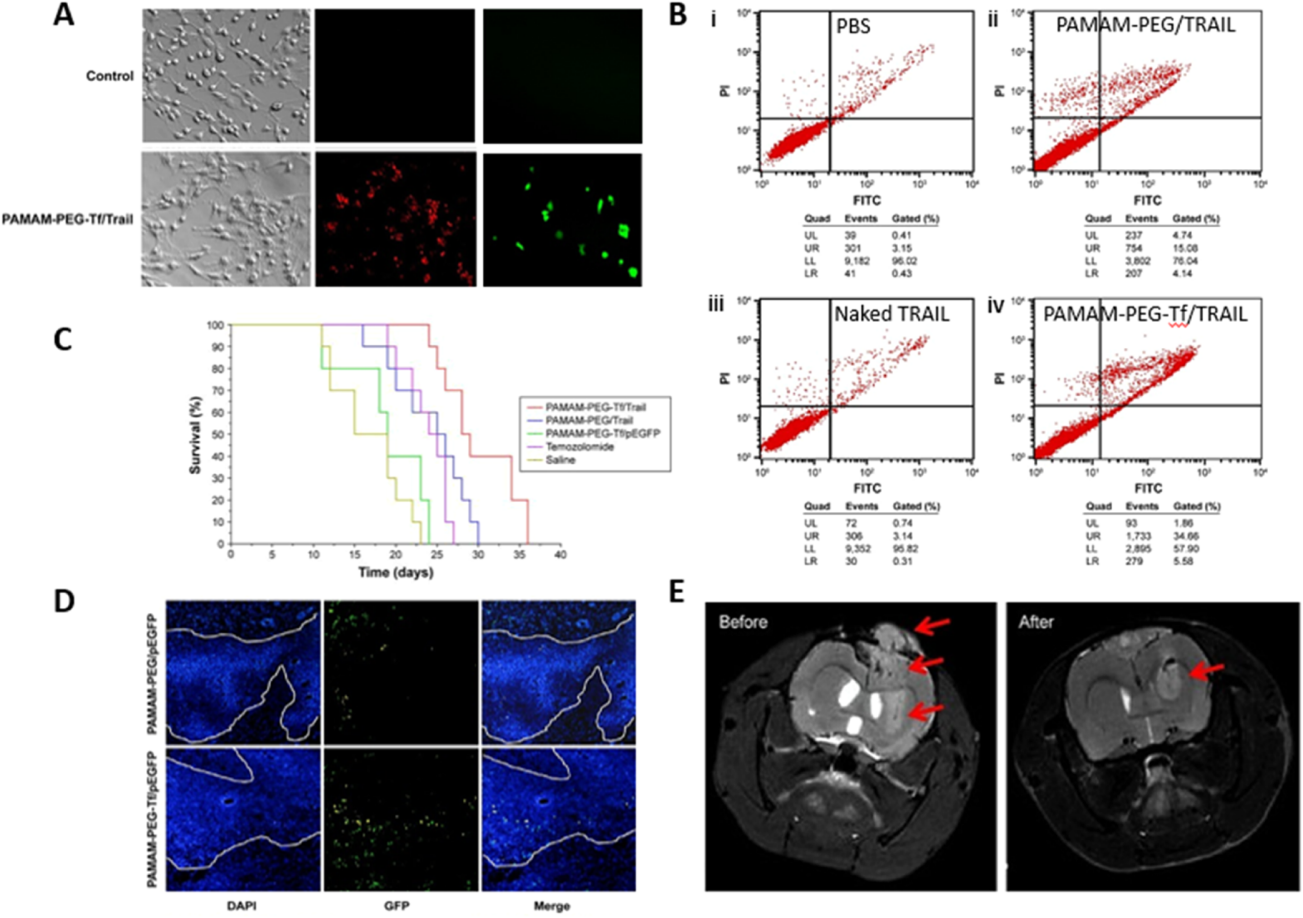
PAMAM–PEG-Tf/Trail dendrimers efficiently target glioma for gene therapy. (a) Uptake and gene expression of the dendrimers in C6 glioma cells in vitro; the red signal is EMA-labeled TRAIL, whereas the green signal is GFP. (b) Flow cytometry data showing cell apoptosis in vitro. (c) Survival plot of C6-bearing rats in vivo, *n* = 10. (d) Distribution of gene expression in C6-bearing rats treated with the dendrimer. (e) MR imaging of a C6-bearing rat brain, with a red arrow indicating decreased tumor volume after treatment. Reprinted from Gao et. al, 2015, with permission from Taylor & Francis.

**Figure 5:**
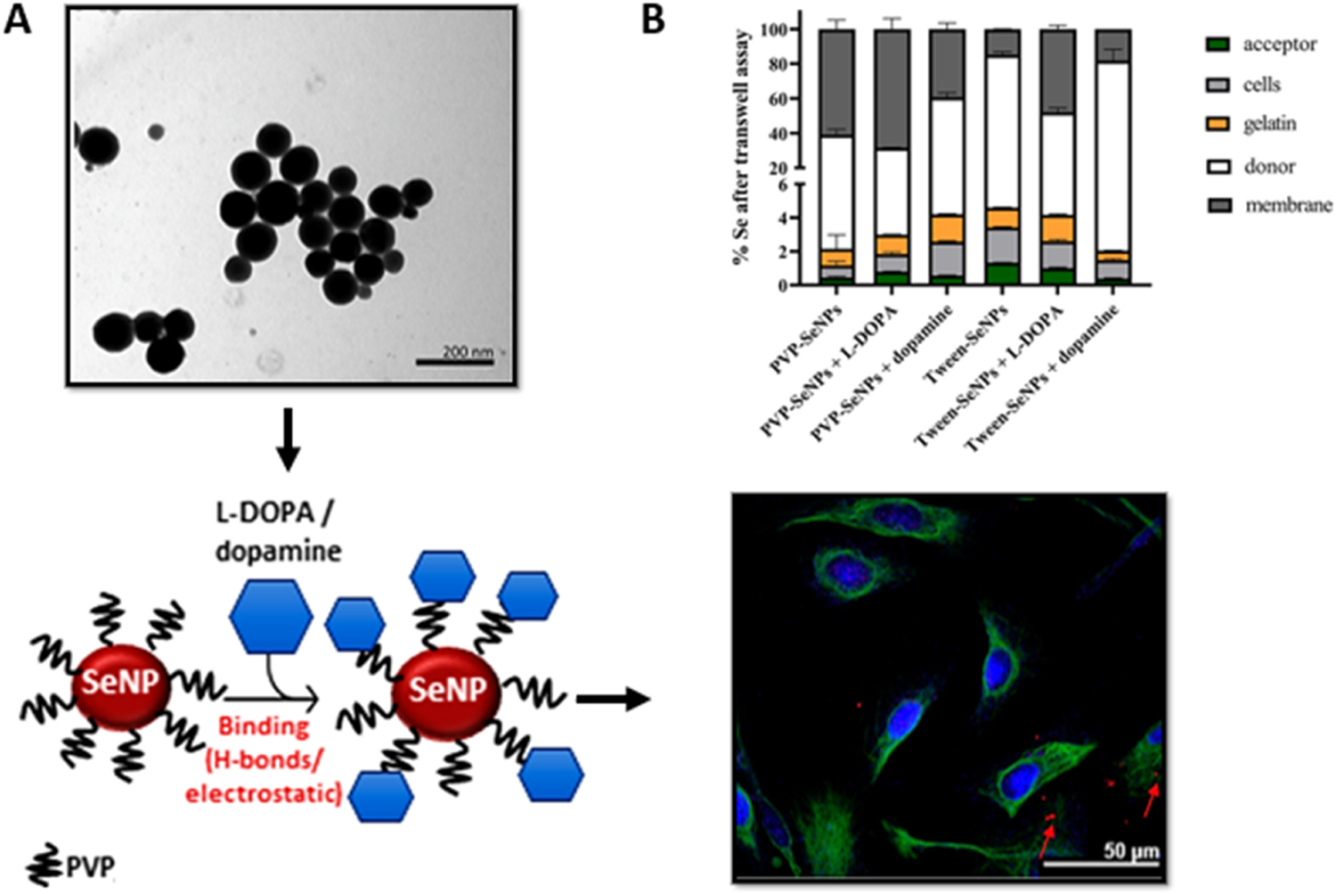
PVP-SeNP-loaded L-DOPA/dopamine, showing high potential for drug delivery and BBB permeability in vitro. (A) TEM image of PVP-SeNPs, representation of PVP-SeNP-loaded L-DOPA/dopamine, and confocal image of hBEC-5i cells treated with PVP-SeNP-loaded L-DOPA/dopamine, showing its ability to cross the BBB in vitro. (B) Se presence in acceptor, cells, gelatin, donor, and membrane, determined via Transwell assays in vitro. Reprinted from Kalčec et al., Copyright American Chemical Society (2015).

##### 1.4.2.4 Polymersomes

Polymersomes, such as lipids, are amphiphilic compounds that contain hydrophilic (water-loving) and hydrophobic (water-repelling) components. These amphiphilic qualities enable self-assembly into vesicles in water, with hydrophobic blocks clustering to avoid water and hydrophilic blocks facing the surrounding solution. Whereas lipids are naturally generated and biocompatible, thus making them ideal for drug delivery applications, liposomes have drawbacks such as uncontrolled release, biodistribution, and systemic toxicity.^115,153^ These particles resemble liposomes but have a thicker bilayer membrane that can be PEGylated; consequently, they are more stable than liposomes and have enhanced blood circulation capability.^154^ Polymersomes can hold more hydrophobic medicines than liposomes, because of their larger apolar compartments. Preparing polymeric carriers for efficient transport across the BBB into the brain is highly beneficial for CNS medicines, which are often hydrophobic. Georgiva et al. have created a polymersome nanocarrier containing a small dodecamer peptide (1645 g mol^–1^) that successfully crosses the BBB in vitro and in vivo. Phage display was used to identify the peptide G23, which targeted the ganglioside GM1.^155^ The G23 peptide, known for binding gangliosides, has been used to increase nanocarrier transport across the BBB.^156^ One study designed a penetrating-targeting polymersome to enhance transport across the BBB/BTB and targeting of glioma cells, thereby improving treatment efficacy in tumor-bearing mouse models. This polymersome was used to encapsulate Dox, and was conjugated with des-octanoyl ghrelin and folate as a penetrating-targeting carrier to improve BBB/BTB transport and tumor accumulation. The des-octanoyl ghrelin ligand is crucial for transporting polymersomal Dox across the BBB/BTB, and folate aids in the targeting of glioma cells. With bifunctional ligands on the surface of polymersomal Dox, BBB/BTB transport and glioma growth suppression have been found to significantly improve, owing to the synergistic action of two separate endocytosis pathways via des-octanoyl ghrelin and folate.^157^

#### 1.4.3 Metal-Based Nanoparticles

##### 1.4.3.1 Gold Nanoparticles

In recent decades, the study of gold NPs (AuNPs) has attracted interest for their potential implications in nanomedicine, particularly in brain-targeting and delivery.^116^ AuNPs have a promising ability to traverse the BBB, exhibiting greater efficacy in targeting the brain than numerous other delivery strategies. Nevertheless, the use of these tools remains restricted, and further investigation is needed to expand and optimize their ability to cross barriers. AuNPs have exceptional light absorption and scattering capabilities, and therefore are highly valuable in applications such as early identification of cancer cells through tumor imaging. This ability is anticipated to induce the movement of electrons on the surface of the metal, as well as the collective oscillation known as surface plasmon resonance, thus leading to stimulation by light at specific wavelengths.^158^ For example, versatile AuNPs coated with the chemotherapy drug cisplatin have been developed to enhance radiation sensitivity. Cisplatin is effectively transported to the brain; moreover, gold and platinum atoms can also absorb the high-energy radiation emitted by electrons and subsequently generate cytotoxic reactive oxygen species. This process enhances the in vivo cytotoxicity and therapeutic effectiveness of this NDS for malignant brain tumors.^159^ One clinical trial has examined the safety of NU-0129, an innovative drug based on spherical nucleic acid technology, in patients with recurrent GBM or gliosarcoma. NU-0129 is a compound composed of nucleic acids bound to a tiny spherical gold NP to cross the BBB. Once inside the tumor, NU-0129 targets the Bcl2L12 gene, which has been associated with tumor growth and prevents programmed cell death (apoptosis). Blocking this gene is expected to halt the growth of cancer cells. This study is the first human trial of NU-0129 to determine its safety (NCT03020017).

##### 1.4.3.2 Magnetic Nanoparticles

Magnetic NPs use a magnetic field gradient to deliver drugs, and are typically based on metal elements such as iron, nickel, and cobalt. The strong field irreversibility and high saturation field of these magnetic NPs offer potential benefits. In addition, a significant superparamagnetic presence is characterized by the occurrence of additional anisotropy contributions or shifted loops during field cooling.^117,160^ Typically, biodegradable polymers are used to encase magnetic NPs, and the effectiveness of these NPs greatly relies on the material used for brain cancer treatment. Cobalt and nickel are extremely susceptible to oxidation. Magnetic NPs have higher toxicity than iron oxide compounds. The ideal candidates for this form of nanomedicine are individuals with high surface areas, diminished sedimentation, and elevated tissular diffusion. Magnetic NPs provide unique benefits for delivering drugs to the brain: magnetic NPs with enhanced surface effectiveness significantly decrease magnetic dipole–dipole interactions. The location of the delivered drug can be adjusted depending on the equilibrium between the magnetic forces and the forces exerted by the blood compartment.^117^ Magnetic NPs are also useful in imaging brain tumors. Gadolinium compounds, which are rare earth metals, have been successfully used in MRI scanning to detect NPs that accumulate in tumor areas.^161^

##### 1.4.3.3 Microbubble Nanoparticles

Microbubble NPs are theragnostic agents with a unique ability to interact with ultrasound. This characteristic makes them highly valuable in various biological and medical applications. Every microbubble NP consists of a gas core enveloped by proteins, lipids, or polymers. Molecular imaging uses the sensitive effects of the acoustic backscattering of light rays. This method shows significantly higher echogenicity than conventional ultrasonography. Lower acoustic pressure induces steady oscillation, thereby increasing the permeability of tight junctions in the BBB to allow penetration of NPs. Under high acoustic pressure, the microbubble initiates a process of splitting into smaller bubbles known as daughter bubbles.^120,162^ The process of breaking microbubbles into smaller fragments has a wide range of practical uses in medical applications. One example is the use of the inertial cavitation technique to deliver drugs specifically to tumors during therapy. These microbubbles are designed with chemical compositions to facilitate targeted drug delivery. Protein shells, surfactant shells, lipid shells, polymer shells, and polyelectrolyte multilayer shells are among the many types of shells used to construct microbubbles to provide several advantages. Because of its ultrasound functionality and ability to cross the BBB, made them important for studies.^120^

##### 1.4.3.4 Quantum Dot Nanoparticles

QDs are semiconductor nanomaterials with dimensions in the nanometer range. QDs can be constructed from a variety of materials, including metals (such as gold), carbon-based materials (such as carbon dots and graphene), and semiconductors (such as selenium and cadmium). These nanoscale particles have high intrinsic luminescence, and their distinctive quantum phenomena, which occur within a narrow size range, confer distinct advantages in their optical properties.^163,164^ Graphene quantum dots (GQDs), carbon quantum dots (CQDs), carbon nanodots, and carbonized polymer dots are distinct types of QDs (Figure 6). Their differences result from various levels of carbonization, graphitization, and polymerization throughout the synthesis process. QDs have various remarkable optical features, including extraordinary resistance to photobleaching, a high absorption cross-section, and relatively long fluorescence lifetimes.^165^ The optical and electrochemical features of QDs make them ideal for biophotonic and nanomedicine research applications. One study has evaluated the specificity and efficacy of QDs complexed with MMP-9-siRNA (matrix-degrading metalloproteinase nanoplex) in downregulating the expression of the MMP-9 gene in BMECs, which constitute the BBB. Our findings have demonstrated that suppressing MMP-9 gene expression increases levels of ECM proteins, including collagen I, IV, and V, and decreases endothelial permeability, as indicated by an increase in the TEER value in a well-established in vitro BBB model. Furthermore, silencing of the MMP-9 gene increases the expression of tissue inhibitor of metalloproteinase-1 (TIMP-1), thereby emphasizing the importance of the balance between MMP-9 and its natural inhibitor TIMP-1 in preserving basement membrane integrity. These findings highlight the potential for this QD-based siRNA delivery technology to modulate MMP-9 activity in BMECs and other MMP-9-producing cells. This technique provides a promising framework for developing therapeutically meaningful QD formulations aimed at avoiding neuroinflammation and maintaining BBB integrity.^166^ Since the discovery of the transferrin receptor protein’s high localization on the brain’s endothelial surface, transferrin has been used to facilitate receptor-mediated transport across the BBB. In a recent study, a transferrin-conjugated quantum dot (Tf-QD) formulation has been developed and used to traverse the in vitro BBB model through receptor-mediated transport mechanisms. Fluorescence analysis of the lower medium revealed that some QD-Tf bioconjugates successfully crossed the BBB. Furthermore, confocal microscopy demonstrated QD staining on both the upper and lower sides of the PET membrane after treatment with the Tf-QD bioconjugates, thus indicating effective translocation of the functionalized QDs. These results support that QDs can be efficiently guided to traverse the in vitro model BBB.^167^ Patel and Shah et al. have conducted both in vitro and in vivo studies to generate water-soluble two-dimensional fluorescent GQD nanocrystals by using bottom-up methods and a simple one-pot synthesis procedure. Spectrofluorimetry, gel electrophoresis, FT-IR, and DLS investigations have demonstrated that carbodiimide-activated amidation achieves a stable combination between GQDs and antibodies/proteins than PEGylation. In silico molecular docking has predicted frequent interactions during this conjugation. In vitro testing revealed dose-dependent toxicity of GQDs and their conjugates, including deadly hemolytic effects, whereas in vivo investigations demonstrated that elevated CRP levels did not cause inflammation in rats. Ethylnitrosourea led to modest brain tumor development, whereas treatment with the GQD-Caspase 8 compound displayed considerable antitumor and neuroprotective benefits in these mice.^168^ In recent years, NPs, particularly CDs, have been demonstrated to be a viable drug delivery approach for CNS diseases. Another in vitro/in vivo study has developed new carbon dots (MGA-CDs) for glioblastoma treatment with metformin and gallic acid as precursors. MGA-CDs have high BBB permeability and strong antitumor activity. They successfully target tumor cell mitochondria without requiring additional targeting agents, thus resulting in mitochondrial shrinkage and decreased cristae. Transcriptome profiling has indicated that MGA-CDs impair the glycerophospholipid metabolic pathway by suppressing PLPP4 expression and consequently result in ferroptosis. Their therapeutic efficacy has been confirmed in a human-derived orthotopic glioblastoma mouse model, wherein MGA-CDs markedly decreased tumor development and increased survival. This study emphasizes the potential of CD-based medicinal compounds for glioblastoma treatment.^169^

**Figure 6:**
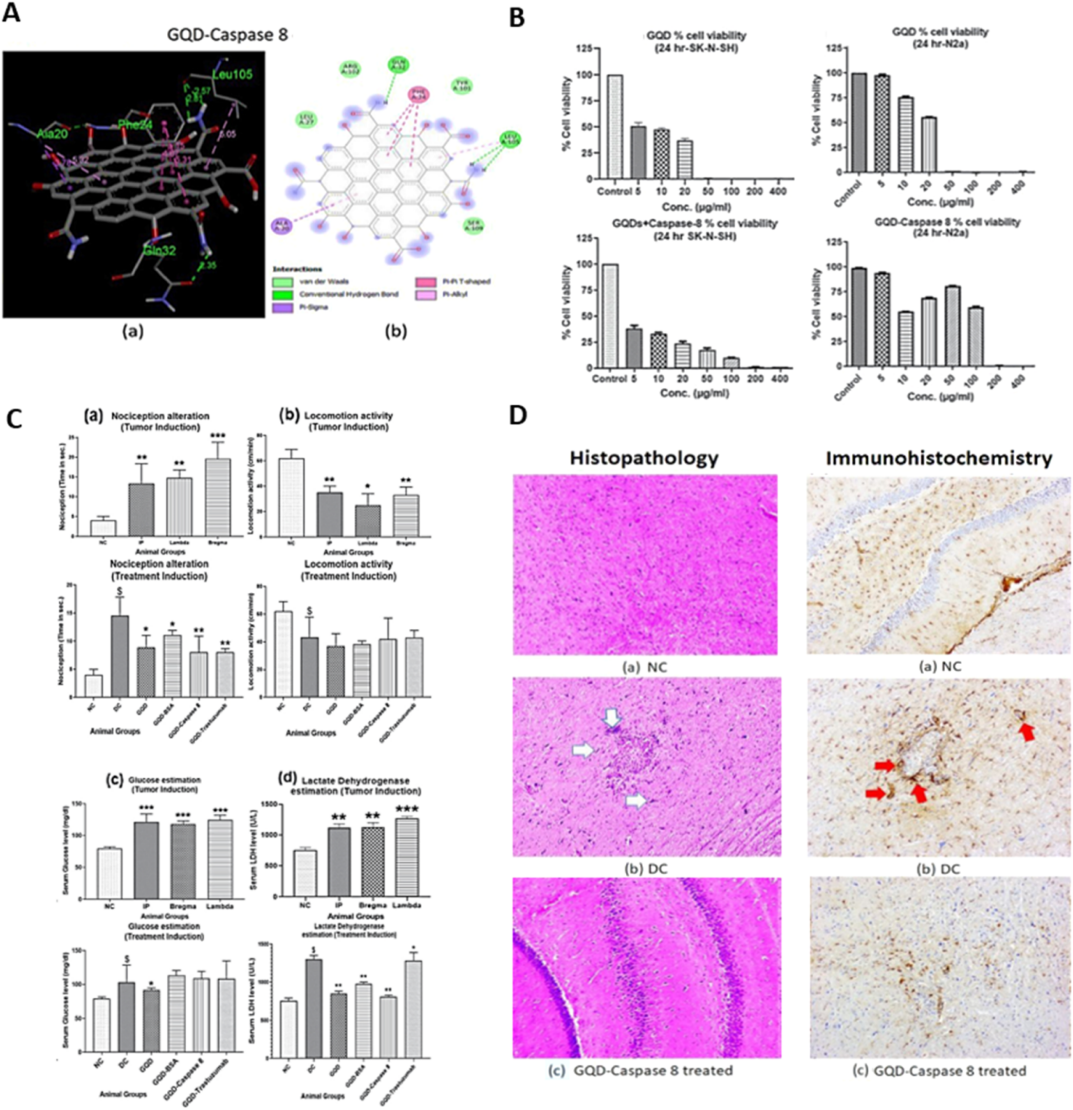
GQD conjugated with Caspase 8 decreases neurodegeneration and demonstrates anticancer behavior against GBM in vivo. (A) Representation of the GQD-Caspase 8 interaction. (B) In vitro cell viability of SK-N-SH and N2a cells 24 h after treatment with GQD and GQD-Caspase 8 separately, with increasing concentrations of NPs. (C) In vivo brain tumor diagnostic biomarker estimation in brain tumor-bearing rats treated with various combinations of GQDs. (D) Histopathological and immunohistochemical characterizations of brain tissues of GQD-treated brain tumor-bearing rats. Reprinted from Patel and Shah, 2023, with permission from IOP Publishing.

##### 1.4.3.5 Mesoporous Silica Nanoparticles

Mesoporous silica nanoparticles (MSNs), which are homogeneous mesopores with simple functionalization and high biocompatibility, have recently gained in popularity for biomedical applications.^119,170^ The pore chambers and vast surface area provide a favorable foundation for creating multifunctional theragnostic agents. The unusual architecture of MSNs enables functionalization of three separate domains: the silica framework, nanochannels/pores, and the nanoparticle’s outermost surface. With various domains for (1) the contrast agent that allows traceable imaging of theragnostic target, (2) the drug payload for therapeutic intervention, and (3) the biomolecular ligand for highly targeted delivery, MSNs are particularly well-suited to combining the fundamental functions of a theragnostic platform in a single particle. Beyond these characteristics, MSNs are readily taken up by cells, enable simple surface functionalization, and have in vivo biocompatibility.^171–173^ In a study, researchers evaluated MSNs with various forms and surface coatings have been evaluated for possible application as brain drug carriers. Spherical and rod-shaped MSNs (less than 100 nm) have been evaluated with or without PEG–PEI coatings. The coated particles demonstrated improved cellular absorption and were safe at the tested amounts. Permeability tests revealed low transport rates across a BBB model. Two-photon in vivo imaging revealed that the MSNs were detectable in the brain vasculature without inducing BBB disruption.^174^ Another study has created ligand-free PEGylated MSNs (RMSN25-PEG-TA) 25 nm in size and with a slight positive charge, which showed enhanced BBB penetration. Two-photon imaging demonstrated that these nanoparticles remained in the circulation for more than 24 h and successfully crossed the cerebrovascular area. When loaded with DOX, the nanoparticles (DOX@RMSN25-PEG-TA) showed 6-fold greater brain accumulation than free DOX, owing to the improved permeability and retention effects. In vivo investigations demonstrated considerable decreases in glioma growth and increases in the lifespan by more than 28% in brain tumor models, as well as improved biosafety. LC-MS/MS revealed a distinct protein corona containing apolipoprotein E and albumin, which is likely to contribute to BBB penetration.^175^

## 2 Conclusions and Future Perspectives

In the CNS in the human body, the BBB is an important membrane that surrounds the blood vessels of the brain and resists the exchange of molecules and/or substances between the blood and the brain.

NDDs are not effectively treated because of the inability of drugs to penetrate the BBB. For an administered medicine to exert effects at the intended location, it must initially traverse this exceptionally selective barrier. Research and testing remain ongoing to identify medicines that are sufficiently small to cross this barrier. Conditions such as AD currently lack treatments, because drug molecules are unable to penetrate the BBB. Hence, nanotechnology may play a crucial role in addressing CNS-associated illnesses by facilitating the targeted delivery of drugs to the brain.

Drug delivery systems comprise several types of polymeric NPs, liposomes, dendrimers, and other such components. These nanocarriers can deliver drugs to specific locations while protecting against enzymatic breakdown, thus contributing to a decreased immune response, improved stability, solubility in the bloodstream, and controlled and extended drug absorption. Additionally, these nanocarriers are sufficiently small to cross the BBB and avoid elimination by the reticuloendothelial system. A deeper understanding of the mechanisms of NPs would facilitate the development of novel therapeutic approaches for brain diseases, thereby overcoming a current limitation in research on CNS conditions. Furthermore, by specifically targeting the drug to the cerebral circulation, increased absorption of a compound by the brain can be achieved while minimizing toxicity to other organs in the body. To facilitate the administration of drugs, nanocarriers must critically maintain stability within the bloodstream, be able to overcome renal clearance and plasma protein binding, and cross the BBB. Hence, a greater understanding of the interactions among numerous factors associated with NP structure would enable the development of more targeted and effective drug delivery systems for the efficient transport of therapeutic drugs and diagnostic compounds across the BBB. In summary, nanocarriers may significantly contribute to the treatment of CNS diseases. The potential of these nanocarriers in applications for the treatment of CNS-associated illnesses and their ability to penetrate the BBB to deliver therapeutic amounts of drug are highly encouraging.

## §

Department of Mechanical Engineering, Marmara University, Maltepe, 34854 Istanbul, Turkey
